# The complete mitochondrial genome of the Hainan Glass Lizard (*Dopasia hainanensis*) determined by next-generation sequencing

**DOI:** 10.1080/23802359.2019.1700194

**Published:** 2019-12-12

**Authors:** Bo Cai, Xianguang Guo, Zhaobin Song, Dali Chen

**Affiliations:** a Chengdu Institute of Biology, Chinese Academy of Sciences, Chengdu, Sichuan, China;; b College of Life Sciences, Sichuan University, Chengdu, Sichuan, China;; c University of Chinese Academy of Sciences, Beijing, China;; d Key Laboratory of Bio-Resources and Eco-Environment of Ministry of Education, College of Life Sciences, Sichuan University, Chengdu, Sichuan, China;; e Department of Parasitology, West China School of Basic Medical Sciences and Forensic Medicine, Sichuan University, Chengdu, Sichuan, China

**Keywords:** Anguidae, Hainan Glass Lizard, mitochondrial genome, next-generation sequencing, phylogenetic tree

## Abstract

The complete mitochondrial genome of the Hainan Glass Lizard (*Dopasia hainanensis*) from its type locality (Diaoluo Mountain in Hainan Island, China) was determined using next-generation sequencing. The mitogenome was 17,000 bp in length and comprised the standard set of 1 control region, 2 rRNAs, 22 tRNAs, plus 13 protein-coding genes (PCGs). The PCGs were used to perform Bayesian phylogenetic analysis together with other Anguidae and Helodermatidae as well as Shinisauridae lizards with mitogenome in GenBank. The resulting phylogenetic tree recovered *D. hainanensis* as the sister-taxon to *Dopasia harti*. The mitogenome of *D. hainanensis* will provide a valuable resource for various study areas such as species delimitation, molecular evolution, and phylogenetic inference.

The glass lizards of genus *Dopasia* represent a group of legless anguid lizards, comprising seven species distributed from northern India through the Indochinese peninsula southwards to Indonesia (Nguyen et al. 2011; Uetz et al. 2019). Currently, there are three species of *Dopasia* occurring in China (Cai et al. 2015). Among them, the Hainan Glass Lizard, *Dopasia hainanensis*, is endemic to the Indochina subregion, including Hainan Island (Yang 1983) and Vietnam (Nguyen et al. 2011). So far, little is known about the mitochondrial genome (mitogenome) evolution in *Dopasia* lizards, even in Anguidae, or Alligator Lizards.

In this study, we report the whole mitogenome of *D. hainanensis* determined by next-generation sequencing. The specimen (field number CB2018037) was collected from the type locality of Diaoluo Mountain, Hainan Island, China on 31 July 2018. Its liver tissue was fixed with 95% ethanol and stored at −20 °C in the herpetological collection, Chengdu Institute of Biology, Chinese Academy of Sciences. A small amount of liver tissue was shipped to Tsingke (Chengdu, China) for genomic extraction and 150-base-pair paired-end library construction; sequencing was performed on an Illumina Hiseq 2000 instrument (Illumina, San Diego, CA). *De novo* assembly of clean reads was performed using SPAdes v3.11.0 (Bankevich et al. 2012). The mitogenome of *Dopasia harti* (GenBank accession number KF279681; Pan et al. 2015) was further used as a reference to assemble that of *D. hainanensis*. Genes were annotated with the MITOS web server (Bernt et al. 2013).

The mitogenome of *D. hainanensis* is 17,000 bp in length, comprising 13 protein-coding genes (PCGs), 22 tRNA genes, 2 rRNA genes, and a control region (CR or D-loop). The gene organization and order exhibited a typical vertebrate mitogenome feature. Most genes were encoded on H-strand except for *ND6* and eight tRNA genes (*tRNA-Gln*, *Ala*, *Asn*, *Cys*, *Tyr*, *Ser^UCN^*, *Glu*, and *Pro*). Most PCGs were initiated with the typical ATG codon, except for *COX1* with GTG and *COX3* with ATA. Meanwhile, most PCGs were terminated with the typical TAA/TAG/AGG codons, except for *COX2*, *COX3*, *ND3*, and *ND4* with the incomplete termination codon T. The 22 tRNA genes ranged in size from 64 bp in *tRNA-Lys* and *tRNA-Ser^AGY^* to 73 bp in *tRNA-Leu^UUR^* and *tRNA-Asn*. The *12S rRNA*, *16S rRNA*, and *D-loop* were 944, 1554, and 1555 bp in length, respectively.

To evaluate the mitochondrial sequence authenticity of *D. hainanensis* and its phylogenetic placement, we performed phylogenetic analysis using eight mitogenomes of the anguid lizards and *Heloderma suspectum* (Helodermatidae) plus *Shinisaurus crocodilurus* (Shinisauridae) as outgroup taxa. We concatenated the PCGs manually and performed Bayesian inference with MrBayes v.3.2.1 (Ronquist and Huelsenbeck, 2003) with GTR + G + I model of substitution. The phylogenetic tree (Figure 1) indicated a close phylogenetic affinity of the congeners and confirmed *D. hainanensis* as the sister-taxon to *D. harti* (Lavin and Girman 2019). The results also recovered the monophyly of Anguidae and Anguinae (Lavin and Girman 2019; and references therein). The mitogenome of *D. hainanensis* will provide a valuable resource for various study areas such as species delimitation, molecular evolution, and phylogenetic inference.

## Nucleotide sequence accession number

The complete mitochondrial genome sequence of *Dopasia hainanensis* has been assigned GenBank accession number MN640999.

## Figures and Tables

**Figure 1.**
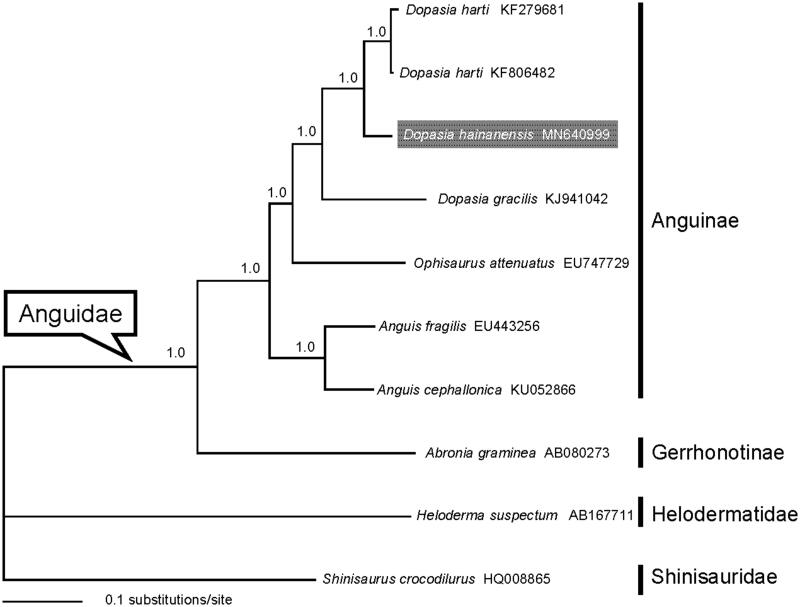
A majority-rule consensus tree inferred from Bayesian inference using MrBayes v.3.2.2 (Ronquist et al. 2012) under the GTR + G + I substitution model, based on the concatenated PCGs of eight lizards of Anguidae and two outgroup taxa representing Helodermatidae and Shinisauridae. The sequenced sample was highlighted in gray. DNA sequences were aligned in MEGA v.6.06 (Tamura et al. 2013). The PCGs were translated into amino acids sequences, and were manually concatenated into a single nucleotide dataset (in total 11,492 bp). Node numbers show Bayesian posterior probabilities. Branch lengths represent means of the posterior distribution. GenBank accession numbers are given with species names.
